# Structure Optimization of Some Single-Ion Conducting Polymer Electrolytes with Increased Conductivity Used in “Beyond Lithium-Ion” Batteries

**DOI:** 10.3390/polym16030368

**Published:** 2024-01-29

**Authors:** Dan Butnicu, Daniela Ionescu, Maria Kovaci

**Affiliations:** 1 Department of Basics of Electronics, Faculty of Electronics, Telecommunications, and Information Technologies, “Gheorghe Asachi” Technical University of Iasi, Carol I Blvd, No. 11, 700506 Iasi, Romania; dbutnicu@etti.tuiasi.ro; 2 Department of Telecommunications and Informational Technologies, Faculty of Electronics, Telecommunications, and Information Technologies, “Gheorghe Asachi” Technical University of Iasi, Carol I Blvd, No. 11, 700506 Iasi, Romania; 3 Department of Communications, Faculty of Electronics, Telecommunications, and Information Technologies, “Politehnica” University of Timisoara, V. Pârvan Blvd., No. 2, 300223 Timisoara, Romania

**Keywords:** beyond Li-ion batteries, gel polymer electrolyte, conductivity, dopant, simulation, modified coarse-grained molecular model, double-parametrical analysis

## Abstract

Simulation techniques implemented with the HFSS program were used for structure optimization from the point of view of increasing the conductivity of the batteries’ electrolytes. Our analysis was focused on reliable “beyond lithium-ion” batteries, using single-ion conducting polymer electrolytes, in a gel variant. Their conductivity can be increased by tuning and correlating the internal parameters of the structure. Materials in the battery system were modeled at the nanoscale with HFSS: electrodes–electrolyte–moving ions. Some new materials reported in the literature were studied, like poly(ethylene glycol) dimethacrylate-x-styrene sulfonate (PEGDMA-SS) or PU-TFMSI for the electrolyte; p-dopable polytriphenyl amine for cathodes in Na-ion batteries or sulfur cathodes in Mg-ion or Al-ion batteries. The coarse-grained molecular dynamics model combined with the atomistic model were both considered for structural simulation at the molecular level. Issues like interaction forces at the nanoscopic scale, charge carrier mobility, conductivity in the cell, and energy density of the electrodes were implied in the analysis. The results were compared to the reported experimental data, to confirm the method and for error analysis. For the real structures of gel polymer electrolytes, this method can indicate that their conductivity increases up to 15%, and even up to 26% in the resonant cases, via parameter correlation. The tuning and control of material properties becomes a problem of structure optimization, solved with non-invasive simulation methods, in agreement with the experiment.

## 1. Introduction

The “beyond lithium-ion” batteries implemented with single-ion conducting polymer electrolytes (SIPEs) present a higher energy density when the materials for electrolyte, electrodes, and moving ions are properly chosen. This represents a complex task, due to the fact that these materials are strongly correlated, and internal interactions occur at the molecular level [1,2,3].

The solid SIPEs have no more disadvantages like electrolyte leakage or flammability, which are characteristics of batteries with liquid electrolytes. For these conducting polymers, anions are fixed to the polymeric chain and only cations are capable of migrating in the structure, from one chain to another, but bind and unbind with the chains by weak molecular forces. Thus, only one type of ion, the cation, participates in the electrical conduction inside the battery cell.

This behavior presents some advantages, namely that concentration polarization does not appear in the SIPEs with immobile anions, which implies the absence of cation depletion. For this type of SIPEs, the Sand and diffusion coherences are invalid, and cation conduction occurs by migration and not by diffusion, like in the dual-ion batteries [4,5,6].

Usually, electrodes chosen for different categories of electrolytes can be an insertion or a conversion material type. In the case of SIPE electrodes, the proper choice is represented by the conversion electrode type, usually carbonates or ethereal solvents. Other categories of materials can be an option, which involve bond breakage and significant structural changes in the electrode materials during redox reactions, like light chalcogens, sulfur, and oxygen, considering that the electrode material has to be compatible with the movable cations’ nature.

In the electrode material, redox reactions occur, and the conversion anode stores and releases cations, storing charge via cation replacement in an ionic compound by the active ion. The negative charge state can be stabilized by conjugated structures via charge delocalization [7,8]. The energy density generated by an electrode is directly correlated with the charge capacity and the redox potential of the active species involved. The compatibility between cathode material and movable cation species should be chosen using the periodical system, analyzing valences and redox potential [7]. Redox-active polymers and also carbonyl-containing polymers, particularly polyquinones and polyimides, are also good candidates for electrodes, also in correlation with the polymer electrolyte. These structures present the advantage of higher energy density due to the complex phenomena occurring inside, which facilitates the conductivity increase. In these cases, the possibility of tuning the structural parameters that control the conductivity in the cell is also considerable.

The gel polymer electrolytes are a good option for performance battery cells because a solvent inserted in a polymer helps in ion transport across the polymer matrix. The solvent has to be chosen with a suitable dielectric constant, which is noted to impact ion conductivity. The gel polymers are typically prepared via thermal or UV curing methods by initially impregnating liquid precursors inside the electrode [1,9,10,11]. It is compulsory to apply an in situ polymerization method, which presents the advantage of decreasing the interfacial resistance between the solid electrolyte and electrodes [12,13].

In the new “beyond lithium-ion” batteries, the Li^+^ ions are replaced with cations like Na^+^—which has similar physico-chemical properties to lithium—or with K^+^, Mg^2+^ and Ca^2+^, or Al^3+^. The interest in these materials is for their accessibility, cost, correlation with electrode materials, easy conduction in the electrolyte, etc., all at an even lower cost. The valences, ionic radii, and mobilities of the alternative cations are of interest when the proper electrodes–electrolyte materials are chosen. Different variants are tested in the literature and new phenomenological valences occur when studies are evolving.

Consequently, the purpose of the new SIPE variants for electrolytes is to have an increased conductivity (the goal is a conductivity higher than 10^−3^ S/m), which can be achieved by increasing their chain mobility and tuning the strength of the interaction between the polymer polar groups and the active ion [6,14], which is no more the classical lithium. This represents the main interest of our study, where the idea is to analyze and optimize different SIPE variants.

A non-invasive analysis method, which is very flexible and facilitates the variation in internal and external parameters, is of much interest in practice. The structure optimization for deserving a specific task (e.g., improved conductivity in the case of SIPEs) can be efficiently performed when more than one parameter is varied and this variation has to be correlated. Following this idea, the simulation method of the intimate structure at the molecular level, with fair consideration of the internal interaction between the constituents, can provide us with information about the composite material behavior when different stimuli are applied.

We performed our study for the case of “Beyond lithium-ion” batteries with improved cations conductivity, comprising a gel single-ion polymer electrolyte. The study target was “something else than Lithium cations”, with possibilities of indicating results for new cation species, from an open list, and also “a larger variety of materials for the medium where conduction occurs”, such as, for now, polymers, but the material list is also open.

The internal structure was described by simulation based on the coarse-grained molecular dynamics (CGMD) model, modified for the gel polymer electrolyte. The mesoscopic particle-based model or dissipative particle dynamics (DPD) model has been used for conductivity determination of the phases inside the polymeric gel. The electrolyte conductivity was calculated based on our method and also the electrodes’ energy density. Structure optimization techniques via more-parameter correlation were revealed. The possible resonances corresponding to the conductivity maxima were identified when different structural parameters were correlated. Our new approach consists of physical parameters maximization and discovery of the resonances, by internal parameters correlation, and more than one internal parameter has to be modified to obtain a maximum (e.g., electrical conductivity maxima).

Our study was dedicated to improving the model for the new materials’ analysis for batteries.

## 2. Materials and Methods

For analyzing the “beyond lithium-ion” batteries with SIPE, the structure of interest is represented by the electrolyte–electrodes–moving ions system. These elements are interdependent and the phenomena implied are correlated.

Different variants of structures have been analyzed to find possibilities to increase the conductivity inside. The gel polymer variant is recommended to be better, for which an amount of solvent is incorporated into the SIPE, compatible with the electrode material (carbonates, ethereal solvents). Conversion electrode materials (e.g., halides, light chalcogens) have also been considered and the results for the conductivity in the cell have been compared with those reported in the literature [15,16].

**Figure 1:**
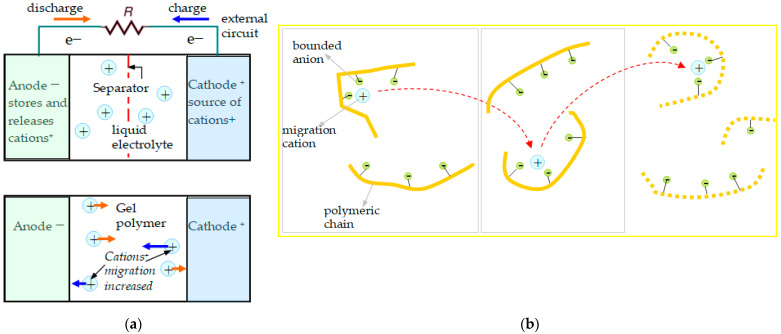
Single-ion conductive polymer working principle in the battery cell. (**a**) Structure of electrodes–electrolyte; (**b**) cation migration in the polymer matrix, from one chain to another; fixed anions can be observed.

The single-ion versus dual-ion conducting electrolytes [5,17] were adopted for analysis. In the dual-ion batteries case, cations have to be inserted into the anode, and at the same time, anions are inserted into the cathode. These cations can be metal ions (Li^+^, Mg^+^, Ca^2+^, Al^3+^, etc.), while the anion is usually the counterion in the salt containing the cation, such as PF_6_^−^ or TFSI^−^, etc. In dual-ion batteries, the internal processes are more complicated due to the two transport ion species inside, but they present the advantage of high energy densities, much easier to obtain in comparison with single-ion electrolytes. This represents no more an obstacle for implementing new variants, as single-ion batteries using polymers follow the tendency to be cheaper, safer, and easier to be synthesized. The cation migration in the single-ion structure is illustrated in Figure 1.

We illustrated our method for a group of specific materials for the electrolyte–electrodes–moving ions system indicated below. The target group of materials was chosen based on different criteria: different complexity of the polymeric chain, without and with crosslinks; capability of transporting different cations; the existence of a previous study about these materials used for battery applications and more; a study performed using different methods from ours (the results were compared to validate our method); the presence of different information in the literature about internal structure identities, at the molecular level, starting with the ions and ending with the polymeric beads, and also information about the strength and characteristics of the internal interaction forces between this entities and with the external stimuli; and not least, the foreseeing of new valences and possibilities of exploiting these materials. The target group was:

- Electrolyte: single-ion conducting polymer: poly(ethylene glycol) dimethacrylate-x-styrene sulfonate (PEGDMA-SS) (denoted (1)) [1], polyurethane-trifluoromethane sulfonamide (PU-TFMSI) (denoted (2)) [11], or polydimethyl siloxane-poly(sodium1-[3-(methacryloyloxy)propylsulfonyl]-1-(trifluoromethane sulfonyl) imide-poly(ethylene glycol) methacrylate)-polydimethylsiloxane(PDMS-poly(MPA-Na^+^-*r*-PEGMA)_20_-PDMS_10_) (denoted (3)) [18]. Polymers were SIPE type, in the variant of gel polymer electrolyte. The considered solvents were: dicloro-methane (DCM, organochlorine) and tetrahydrofuran (THF) in PEGDMA-SS; benzoyl peroxide ((BzO)_2_. organic peroxide) and THF in PU-TFMSI, that is, dimethylformamide (DMF) and THF in PDMS-poly(MPA-Na^+^-*r*-PEGMA)_20_-PDMS_10_, respectively.
- Electrodes: conversion electrodes type, as follows: for sodium-ion batteries: p-dopablepolytriphenyl amine as cathode, n-type redox-active poly(anthraquinonyl sulphide) [13,19] or redox-active organic poly(anthraquinonyl imide)s (PAQIs) [20] as anode [21]; for magnesium-ion batteries: sulfur cathodeMo_6_S_8_ [22,23]; for aluminum-ion batteries: glassy carbon/Co_3_S_4_ cathode [24].
- Moving ions: ions with low electrochemical potential vs. standard hydrogen electrodes: cations: those with different valences and atomic radii, such as Na^+^, Mg^2+^, and Al^3+^.

The molecular structures of the polymer electrolyte clusters are indicated in Table 1. (The polymer systems were gelled in the next stage for obtaining the electrolyte). The PEGDMA-SS conductive polymer was obtained with poly(ethylene glycol) diamethacrylate (PEGDMA) copolymerized with ionic monomer styrene sulfonate (SS), resulting in freestanding crosslinked networks [1]. The crosslinked single-ion polymer contains different components with increased content of PPG-PEG-PPG2700 [11]. The new PU-TFMSI polymer reported in the literature is based on a sulfimide type of single-ion oligomer with a polyether type main chain P-TFMSI-Cation, dual-terminated by hydroxyl groups. This results in a urethane-linked polymer, with fixed pyrrole employing bis(triflouro methane sulfonyl) imide (TFMSI) anion, presenting high ionic conductivity: over 1.3 × 10^−2^ Sm^−1^ [11,15,25]. The third considered polymer, (PDMS-poly(MPA-Na^+^-*r*-PEGMA)_20_-PDMS_10_), is a complex one. The MPA-Na^+^ is a recently developed single-ion conducting monomer, used for the synthesis of this polymer electrolyte. This monomer and PEGMA are grafted from the PDMS backbone to form a grafted block copolymer: PDMS-g-poly(MPA-Na^+^-*r*-PEGMA)_20_. After adding PDMS crosslinker (which represents an elastic polymer backbone), we obtained a freestanding elastic polymer membrane of PDMS-poly(MPA-Na^+^-*r*-PEGMA)_20_-PDMS_10_ [18].

The cathode represents the source of cations in the cell. The chosen conversion cathodes have been reported with high energy density for battery applications [26]. The calculation of the density of charge by applying the conservation of charge law for the electrode system is of interest. The conversion anode stores and releases cations; in fact, it stores charge via cation replacement in an ionic compound via the active ion [27]:

(1) $$M_{a}X_{\beta }+\beta n\cdot A^{+}+n\cdot e^{-}⇿\alpha \cdot M+\beta \cdot A_{n}X$$

where *M* is the cation, *X* is an anion, and *A* is the active ion implied in the reaction.

Phenomenologically, a high ionic conductivity in SIPE can be achieved above the glass transition temperature in different conditions [28,29]. For increasing the ionic conductivity in the cell, the polymeric chain mobility can be increased and the strength of the interaction between the polymer polar groups and the active ion can be tuned when the polymeric chain suffers modifications [30]. The ion solubility has to be increased, but the ions must not become trapped [30].

Considering the internal phenomena, a mixture of ionic and electronic conductivity occurs in the cell, which can be characterized using a simulation model, developed for non-invasive testing. We focused on modeling the current transport and the potential field inside this complex structure of electrodes–electrolyte–conducting ions. We have to apply the charge conservation law for the three-dimensional electrical conduction inside the structure, illustrated by Equation (2):

(2) $$\nabla \cdot i=\frac{\partial i}{\partial x}\cdot \overbrace{x}+\frac{\partial i}{\partial y}\cdot \overbrace{y}+\frac{\partial i}{\partial z}\cdot \overbrace{z}=0;\;\;\;i=-\sigma \nabla V⟺\nabla \cdot \left(\sigma \nabla V\right)=0$$

where *i* represents the electrical current, *σ* is the electrical conductivity, and *ϕ* is the electrical potential. The quantities *i* = *i*(*x*,*y*,*z*), that is, *V* = *V*(*x*,*y*,*z*), respectively, were considered in 3D representation, describing a volumetric conduction in the polymeric gel. The structure was described considering the intermolecular interactions of the micro-components, and at the macroscopic level, we had to take into account the ohmic losses in all the conducting materials (electrolyte, electrodes, and current collectors) and the contact resistance at the pairs of interfaces.

Thus, the Laplace equation was solved. The effective conductivity of the polymeric gel was calculated based on the coarse-grained molecular dynamics (CGMD) model, modified for the gel polymer electrolyte, with details presented in the following. If for electrical potential, an evolution in an interval is considered (e.g., 0–5 V), then Equation (1) interconnects the current with the obtained potential, and the internal parameters of the structure and parametrical evolution of these quantities can be studied. Structural parameters like conducting ions radius or solvent concentration in SIPE were considered for analysis. Equation (1) was graphically solved using the Mathcad program, with the help of which the 3D gradient could be represented like a surface in space and intersected with the (0, 0) coordinates plane. The obtained values for *i*, when different internal parameters were changed, were represented on 3D graphs, which are available for structure optimization.

Each material was described at the molecular level in HFSS and then the proper mesh was set, in agreement with the molecular dimensions. The mesh was set for each particular analysis, due to the fact that the coarse-grained molecular dynamics (CGMD) model combined with the atomistic model was the basis of our simulation method. The model was developed by calculating the implied parameters’ values in the case of a solid-state polymer electrolyte, and then the model was modified for the gel polymer electrolyte case.

The coarse-grained molecular scale was combined in analysis with the atomic scale, meaning an interval of 10^−10^ to 10^−6^ m and for each case a proper mesh. The polymer macromolecules are represented as beads. Each CG bead represents a group of monomers in a whole polymer structure. Internal interaction was considered between atoms in the chain, where the parameters of interest were the bond length, bond angle, and bond dihedral potentials, but a few degrees of freedom were neglected in the chain to simplify the global chain structure, less important for the interactions with other species. Thus, the beads for each cell configuration were selected. We had to consider as well the interactions with other molecular species at interfaces (interactions between beads and beads or between beads and other molecules (firstly, the solvent–polymer interactions).

In our case, the interacting species are electrolytes (polymer), electrodes, and moving ions. The parameters for calculation of these interacting forces are mainly the bead pair distance (*r_ij_*), bond length (*l*_ij_), bond angles (*θ_ijk_*), and dihedral angles (*ψ*_ijk_) (a 3D spatial configuration of the beads was considered).

Considering the polymer beads, the mesoscopic particle-based model or dissipative particle dynamics (DPD) model [31] was used for the conductivity determination of the phases inside the polymeric gel. The CG spheres interact with each other through purely repulsive soft potentials. These interactions between beads can be fine-tuned to capture the macroscopic phenomena on larger time scales. Polymeric coarse-grained beds interact with forces given by [31,32,33], illustrated in Equation (3):

(3) $$F_{ij}^{DPD}=\sum_{j\neq i}F_{ij}^{C}+\sum_{j\neq i}F_{ij}^{D}+\sum_{j\neq i}F_{ij}^{R}$$

where *i* and *j* represent the indices of the two interacting beads, respectively, $F_{ij}^{C}$ are conservative forces (non-bonded forces, electric, elastic, describing the repulsive properties between coarse-grained beads), $F_{ij}^{D}$ are dissipative forces (friction, describing the friction dissipation between the structural system in the simulated bead), and $F_{ij}^{R}$ are random forces (describes Brownian motion at ambient temperature) [29,30].

The most important term in expression (1) is determined by the conservative forces, which can be estimated with an expression as follows (Equation (4)), describing a soft pure repulsive (excluded volume) force through a distance *r_ij_* between beads *i* and *j* [6,33]:

(4) $$F_{ij}^{C}=a_{ij}\omega^{C}r_{ij}e_{ij}\;\;\;;\;\;\;\omega^{C}\left(r_{ij}\right)=\left(1-\frac{r_{ij}}{r_{C}}\right)\;\;\;\;\;if\;\;\;\;r_{ij}\leq r_{C}$$

where *a_ij_* is the maximum repulsion force between beads *I* and *j*; $\omega^{C}\left(r_{ij}\right)$ is the weight function of the conservative force, ranging from 0 to 1, which represents a simple decaying function of the distance related with the cutoff distance *r_C_,* which is the interaction radius on the dimensions of the simulation system, correlated with the simulation mesh; $e_{ij}=\frac{r_{i}-r_{j}}{r_{ij}}$ is the unit vector pointing from particle *j* to particle *i*. *a_ij_* represents the dynamic interaction parameter that contains the physical-chemical information relevant to the atomic group and depends on the type of conservative force (repulsion force in our case) [6,33]. Conservative forces were considered to vanish at distances greater than *r_C_*.

Dissipative forces in expression (1) take into account the effects of viscosity, which slows down the particles’ motion with respect to each other and is given in Equation (5) [6]:

(5) $$F_{ij}^{D}=-\gamma_{ij}\omega^{D}r_{ij}e_{ij}\cdot v_{ij}e_{ij}$$

where *γ_ij_* is the friction coefficient; $\omega^{D}(r_{ij})$ is the weight function of dissipative force; and *v_ij_* = *v_i_* − *v_j_* represents the particles’ relative velocity.

The random forces in expression (1) can be characterized considering the thermal or vibrational energy of the system [6], as it is indicated in Equation (6):

(6) $$F_{ij}^{R}=\sigma_{ij}\omega^{R}r_{ij}\varsigma_{ij}\cdot \Delta t^{-0.5}e_{ij}$$

where *σ_ij_* represents the magnitude of the noise; $\omega^{R}(r_{ij})$ is the weight function of the stochastic force; Δ*t* is the integral time step and *ζ_ij_* is a random number between 0 and 1 which ensure the conservation of the total momentum.

For each considered polymeric gel system, the bead structure was defined and the parameters necessary for interacting forces calculation were estimated (the bead pair distance (*r_ij_*), bonds length (*l*_ij_), bond angles (*θ_ijk_*), and dihedral angles (*ψ*_ijk_)).

An important issue is the crosslink between the polymeric chain structural groups. Each type of crosslink can be described by the model, but the number and density of the crosslinks vary in the same structure depending on different parameters, like synthesis method, synthesis ambient conditions (e.g., temperature, pH, etc.), presence of a secondary adjuvant reactant, interaction forces with the gel, nature of the contact electrodes. During the operating process of the battery, the cations’ dynamic at discharge and the voltage applied at charging can influence also the crosslinks panel. In this case, only a mediated crosslink density, decided after a great number of experiments, can offer us information about the matter. The presence of the crosslinks influences the cations’ mobility inside the SIPEs, but is not the decisional factor. For deciding a result for the quantities describing the cations’ possibilities of moving inside the crosslinked SIPEs, we considered a crosslink density justified by the values of the physical parameters of the polymer, meaning the molecular mass, the volumetric density, the polarity, and the elasticity coefficient.

A calculation of the ionic conductivity was performed using the interaction force inside the polymeric gel. The ions’ mobility, μ(E,t), is decided by the interaction forces calculated above. The conductivity σ=e·p(x,y,z,t)·μ(E,t) where is *e* the elementary charge, *p*(*x*,*y*,*z*,*t*) is the charge carrier concentration, and E is the electric field.

The determined quantities were used as parameters inserted in the HFSS set-up, where the materials have been described. The results for conductivity of the medium components (polymeric gel) were obtained via simulation.

Generally, the electrolyte conductivity can be increased by increasing the chain mobility and tuning the strength of the interaction between the polymer polar groups and the active ion. These structural details can be implemented in the simulation set-up.

The effective conductivity formula that was used here was the general effective medium (GEM) equation [28,34], valuable for a medium composed of conducting and insulating phases inside the polymeric gel and is illustrated by Equation (7):

(7) $$\frac{f(\sigma_{1}^{\frac{1}{\varkappa }}-\sigma_{ef}^{\frac{1}{\varkappa }})}{\sigma_{1}^{\frac{1}{\varkappa }}+A\sigma_{ef}^{\frac{-1}{\varkappa }}}+\frac{\left(1-f)(\sigma_{2}^{\frac{1}{\varkappa }}+\sigma_{ef}^{\frac{1}{\varkappa }}\right)}{\sigma_{2}^{\frac{1}{\varkappa }}+A\sigma_{ef}^{\frac{1}{\varkappa }}}=0$$

where *σ*_1_, *σ*_2_ represent the electric conductivities of the conducting and insulating phase, respectively, and *σ_ef_* is the effective conductivity of the polymeric gel; *A* is a constant that depends on the phases’ nature; the exponent *ϰ* depends on the filler volume fraction *f* and to the grain (beads) shape. The *A* and *ϰ* parameters were calculated based on the results indicated in the literature for particular values of the effective conductivity determined in specific conditions for different polymeric gels and values of similar magnitude order have been used in the formula for new polymer variants until a confirmed result for the effective conductivity was obtained. An iterative process of approximation was applied until the results have been validated. The formula has to be applied for each conduction mechanism in the polymeric gel.

Then the current density *j* = *σ_ef_∙E* was computed. The purpose was also to obtain the energy density of the gel polymer battery [35,36]. This quantity can be estimated with the Formulas (8):

(8) $$E_{g}=\frac{C_{cell}\cdot V_{cell}}{\sum m_{components}};\;m_{cathode}=m_{active\;material}+m_{solid\;electrolyte}+m_{binder}+(m_{carbon\;particles})\;m_{anode}=\left(\frac{N}{P}-1\right)\left(\frac{C_{cell}}{C_{theor}}\right)(\frac{N}{P_{area}}+1)$$

where *C_cell_* is the capacity of the electrode; *V_cell_* is the operating cell voltage; *m_components_* represents the mass of individual components of the electrode; *C_theor_* is the theoretical capacity of anode, *N/P* is the excess anode content in the system, and *N*/*P_area_* is a constant that represents the fraction of excess area of the anode [35,36].

## 3. Results for the Material Parameters

The applied working strategy was:

- Establishing the bead groups in the polymer structure;
- Calculating all the interaction forces based on the theoretical model and using data from reference values like those in the literature;
- Establishing the conduction mechanism;
- Determining the charge carriers’ mobility using the simulation set-up when the electrical stimuli were set;
- Calculating the conductivity in the polymeric gel;
- Calculating the current density;
- Calculating the energy density corresponding to different electrode–electrolyte combinations;
- Three-dimensional graphical representation in function of different parameters: conducting ions radius, solvent concentration in SIPE, energy density generated by the conversion electrode (correlated with the charge capacity and redox potential of the active species involved), strain and stress in the polymeric electrolyte; parameters corresponding to the beads: the bead pair distance, bonds length, bond angles, and dihedral angles; choosing of the simulation mesh; and external parameters: electric field for polymeric gel testing, temperature.

The current density throughout the domain was determined when different internal and external parameters of the exploited battery were varied, like the conducting ions radius, solvent concentration in SIPE, respectively the obtained electric potential. The considered electrode thickness was of 400 micrometers (μm), with particles inside on the order of μm, with deferential particle sizes (~7×), higher dimensions for active material particles, and lower for the binder. The ionic radii for the considered cations were: Na^+^, Mg^2+^, and Al^3+^: 116 pm (1.16 Å), 86 pm (0.86 Å), respectively 67.5 pm (0.675 Å).

The method was initially tested on the usually used conductive polymers (CPs) based on different polyethylene oxide, polycarbonates and polysiloxanes. The obtained results were in good agreement with those presented in the literature [11,14,15,18].

**Figure 2:**
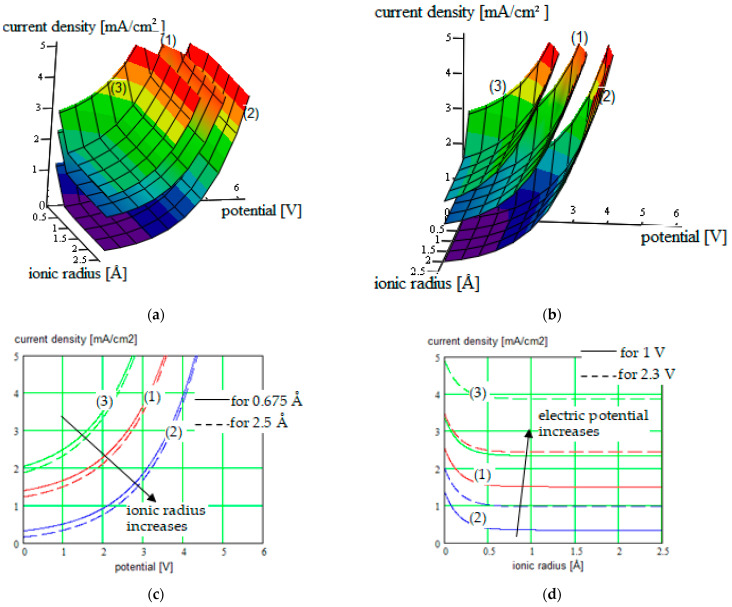
(**a**,**b**) Current density in function of the electric potential, for three species of SIPEs (1), (2), and (3) used for “beyond lithium-ion” batteries electrolyte, considering the ionic radius of the mobile cation like parameter; plots are represented in two positions for clarity; (**c**) Cross-section of the surface plots for constant ionic radius of 0.675 Å (continuous curves), respectively cross-section of the surface plots for constant ionic radius of 2.5 Å (dotted curves), represented on the same 2D graph; (**d**) Cross-section of the surface plots for constant electric potential of 1 V (continuous curves), respectively cross-section of the surface plots for constant electric potential of 2.3 V (dotted curves), represented on the same 2D graph.

**Figure 3:**
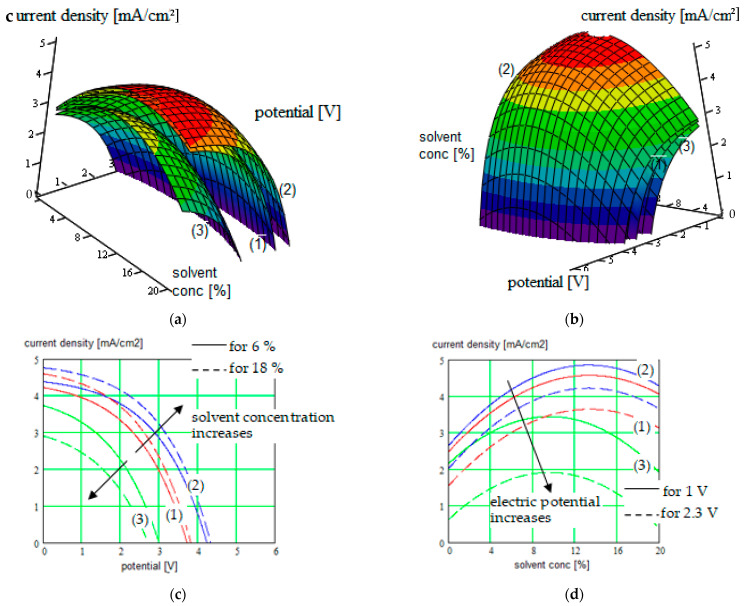
(**a**,**b**) Current density in function of the electric potential for three species of SIPEs (1), (2), and (3) used for “beyond lithium-ion” batteries electrolyte, considering the solvent concentration in SIPE like parameter; plots are represented in two positions for clarity; (**c**) Cross-section of the surface plots for constant solvent concentration of 6% (continuous curves), respectively cross-section of the surface plots for constant solvent concentration of 18% (dotted curves), represented on the same 2D graph; (**d**) Cross-section of the surface plots for constant electric potential of 1 V (continuous curves), respectively cross-section of the surface plots for constant electric potential of 2.3 V (dotted curves), represented on the same 2D graph.

**Figure 4:**
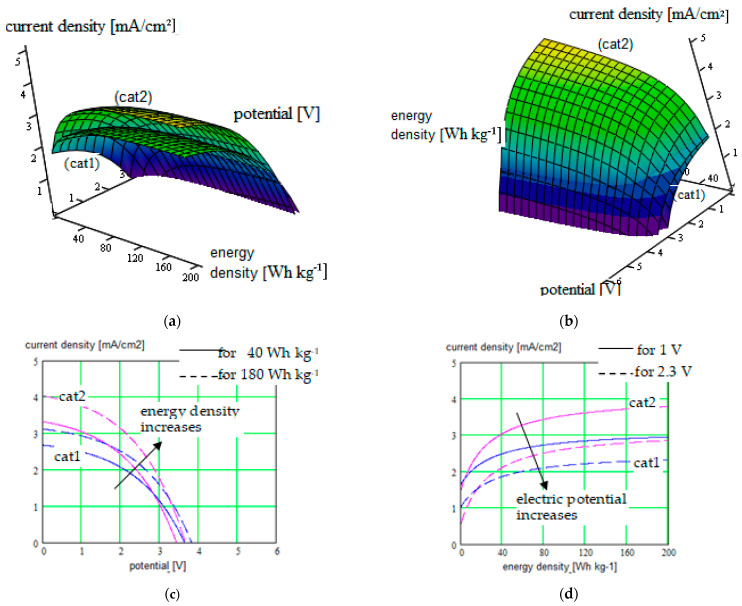
(**a**,**b**) Current density in function of the electric potential for Na-ion batteries, considering the energy density generated by the conversion cathode like parameter. Surface graph (cat1) corresponds to the p-dopable polytriphenyl amine cathode; graph (cat2) is for poly(anthraquinonyl imide) cathode; plots are represented in two positions for clarity; (**c**) Cross-section of the surface plots for constant energy density of 40 Wh kg^−1^ (continuous curves), respectively cross-section of the surface plots for constant energy density of 180 Wh kg^−1^ (dotted curves), represented on the same 2D graph; (**d**) Cross-section of the surface plots for constant electric potential of 1 V (continuous curves), respectively cross-section of the surface plots for constant electric potential of 2.3 V (dotted curves), represented on the same 2D graph.

**Figure 5:**
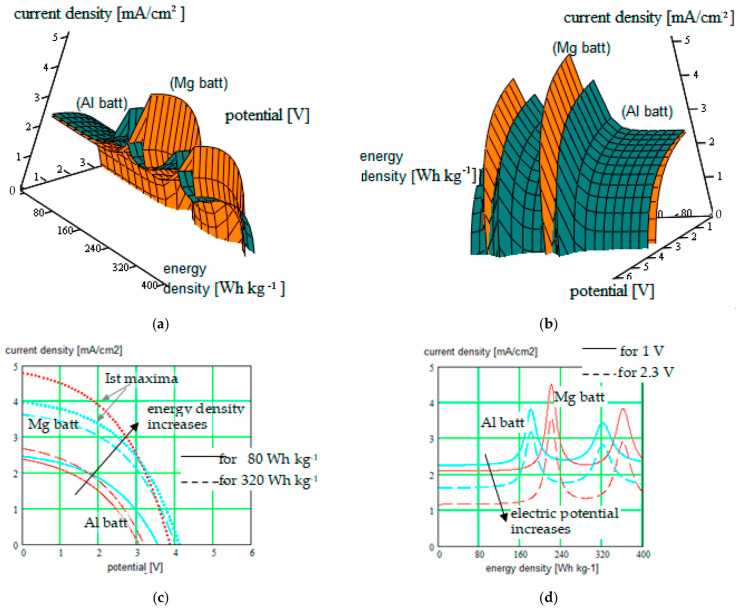
(**a**,**b**) Current density in function of the electric potential for Mg-ion batteries (Mg batt) versus Al-ion batteries (Al batt), considering the energy density generated by the conversion cathode like parameter. The sulfur cathode Mo_6_S_8_ was considered for Mg-ion batteries (orange graph), respectively the Co_3_S_4_ cathode for Al-ion batteries (green graph); plots are represented in two positions for clarity; (**c**) Cross-section of the surface plots for constant energy density of 80 W∙h∙kg^−1^ (continuous curves), respectively cross-section of the surface plots for constant energy density of 320 W∙h∙kg^−1^ (dotted curves), represented on the same 2D graph. Curves for the first maxima, occurring at ca. 220 W∙h∙kg^−1^ for Al batteries, respectively at 182 W∙h∙kg^−1^ for Mg batteries, were also represented on the graph. (**d**) Cross-section of the surface plots for constant electric potential of 1 V (continuous curves), respectively cross-section of the surface plots for constant electric potential of 2.3 V (dotted curves), represented on the same 2D graph.

The new results were obtained for different single-ion CP like (PEGDMA-SS) (1), PU-TFMSI (2) [11], or PDMS-poly(MPA-Na+-*r*-PEGMA)_20_-PDMS_10_(3), but not only (Figure 2, Figure 3, Figure 4 and Figure 5).

The current density versus battery potential were represented on double-parametrical graphs, in order to observe and optimize their dependence on the internal parameters of the structure, characterizing the mobile cations (Na^+^, K^+^, Mg^2+^ and Ca^2+^, Al^3+^) with different valences, the SIPE chain, the solvent incorporated into the SIPE or the conversion electrode. A set of results for the three SIPE mentioned above are illustrated in Figure 2, Figure 3, Figure 4 and Figure 5. Simulations were performed with HFSS program (by Ansys: Ansys HFSS 2022 R1).

One observes that for higher current densities, ionic radius is recommended to be lower, the current density decreasing for bigger cations and also the battery voltage.

One observes that the solvent concentration has an optimum value for which the conduction in the polymer is maximized. Simulations have indicated that this maximum depends in the same time on solvent and movable cation nature.

One observes that a couple of maxima occur on the current density graphs, illustrating internal resonances between a couple of geometrical parameters (conducting ions radius with bead pair distances in our case), which can be identified with the help of the simulation method.

For the solid SIPEs that can be synthesized with increased conductivity, different methods of analysis were reported in the literature. A comparative analysis is imposed in order to reveal the advantages of our method of study. We have considered for illustration the three polymers species analyzed in the 3D parametrical graphs: PEGDMA-SS, PU-TFMSI, and PDMS-poly(MPA-Na^+^-*r*-PEGMA)_20_-PDMS_10_. The comparative analysis is presented in Table 2. We have presented in the table the current density evolution in the gelled SIPEs when different control parameters were varied in specific intervals, imposed by the SIPE nature. In these intervals, the current density of the SIPEs changes, but this property is tuned by the control parameters. These control parameters were enumerated in the table, specific for each analyzing method. One observes that our method offers the possibility of changing the greater number of parameters and their influence to be monitored simultaneously.

## 4. Conclusions

The new SIPE variants present increased conductivity that can be tuned and improved by different methods, like increasing their chain mobility and tuning the strength of the interaction between the polymer polar groups and the active ion, which is a cation that migrates in the structure. These were the basic ideas in our effort to analyze and synthesize different SIPE variants with improved conductivity.

New results were obtained for different single-ion CPs like poly(ethylene glycol) dimethacrylate-x-styrene sulfonate (PEGDMA-SS), PU-TFMSI, or PDMS-poly(MPA-Na^+^-*r*-PEGMA)_20_-PDMS_10_, but not only these. The current density versus battery potential was represented on double-parametrical graphs in order to observe and optimize their dependence on the internal parameters of the structure, characterizing the mobile cations (Na^+^, K^+^, Mg^2+^, and Ca^2+^, Al^3+^), the SIPE chain, the solvent incorporated into the SIPE, and the conversion electrode. A set of results for the three SIPEs mentioned above was illustrated in this paper. The simulations were performed with the HFSS program (by Ansys: Ansys HFSS 2022 R1) based on the modified (CGMD) model for the structural description of the cell constituents.

The conductivity of the gel polymeric electrolyte depends on different parameters: conducting ions radius, solvent concentration in SIPE, energy density generated by the conversion electrode (correlated with the charge capacity and redox potential of the active species involved), strain and stress in the polymeric electrolyte; parameters corresponding to the beads: the bead pair distance, bonds length, bond angles, and dihedral angles; choosing of the simulation mesh; and external parameters: electric field for polymeric gel testing, temperature.

If we consider the matter of an optimal choice of the materials for batteries (SIPEs, gel, and cations) among the analyzed structures, the most important conclusion is that better results for the SIPE conductivity can be obtained only by parameter correlation, e.g., due to the fact that the current density decreases for bigger cations and the battery voltage is important, the Al^3+^ cation with a smaller radius can be a choice. Also, the increased valence number (+3) of a cation offers more possibilities to interact with the bounded anions for increased mobility. Another example: when the solvent is chosen, the solvent concentration has an optimum value for which the conduction in the polymer is maximized, depending on solvent and movable cation nature. The gelled PU-TFMSI (+(BzO)_2_ or THF) can be a choice in this case.

The influence of the cathode material has to be also considered, considering the energy density generated by the conversion cathode. The results illustrated for the Na-ion batteries recommend the poly(anthraquinonyl imide) cathode, with better results, rather than an p-dopable polytriphenyl amine cathode associated with the same cation type.

Another important aspect revealed by our study is the occurrence of the SIPE’s current density maxima, illustrated on some double-parametrical graphs. These maxima are of great interest in practice and their occurrence is an event that has to be caused inside the battery physical system. These maxima can be obtained in results of the internal resonances determined by correlation of specific geometrical parameters (conducting ions radius with bead pair distances in our case), which can be identified with the help of the simulation method. Mg-cation batteries and also Al-cation batteries present these maxima when we adjust the energy density generated by the conversion cathode. Our study performed for more battery constituents revealed the presence of these maxima for every type of cation, but their occurrence is difficult to obtain due to the fact that it implies a very fine correlation of the structural parameters (the phenomenon is a resonant one). From this point of view, the role of the simulation is essential.

The idea of current density maxima opens perspectives and a field for future work in order to identify the possible resonances occurring in the material sample. If these resonances are caused in practice by tuning properly and correlating internal parameters, a consistent increase in conduction can be caused at the structure level.

The simulation non-invasive method is applied like a structure parameters generator for the material chosen in a specific application and helps to set the optimal configuration, when the internal specific interactions are considered, at the molecular level.

## Figures and Tables

**Table 1:** The chemical structure of the molecular clusters (monomers and polymers) for the single-ion conducting polymer electrolytes considered for the analysis.

SIPE	Clusters Chemical Structure
PEGDMA-SS (1)	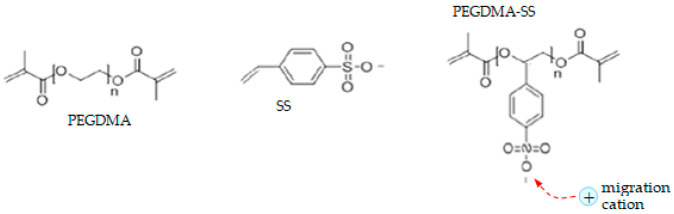
PU-TFMSI (2)	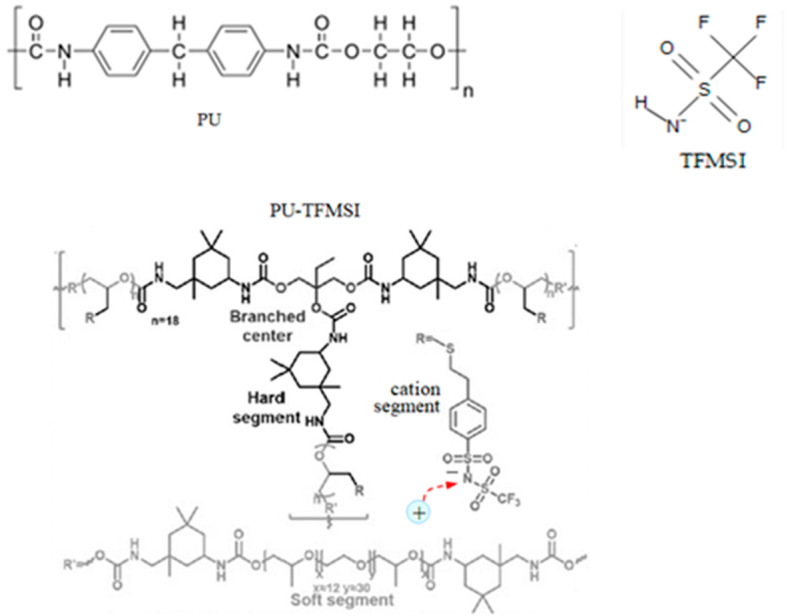
PDMS-poly(MPA-Na^+^-*r*-PEGMA)_20_-PDMS_10_ (3)	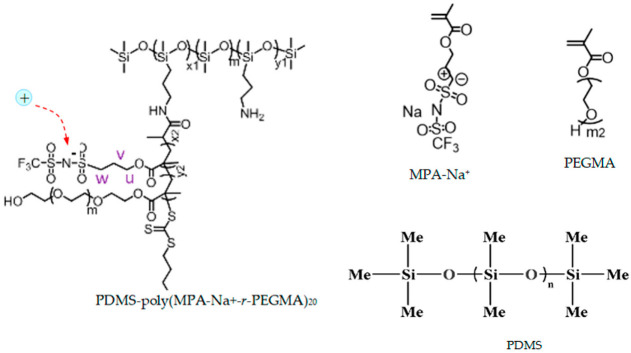

**Table 2:** Method of analysis applied for the current density determination in the “beyond lithium-ion” batteries’ structures with the considered SIPEs and comparison of the results and methods indicated in the literature with our method.

Material: Electrolyte + Solvent	Current or Current Density [mA/cm^2^]/Obtained Electric Potential [V]	Control Parameters	Applications/Characteristics	Method of Determination/Source
/Conversion Electrodes				
**PEGDMA-SS (1) +** diglyme **(DEG),** tetraglyme **(TEG), or** ethylene carbonate + propylene carbonate (**EC:PC)**	*Reported in the literature:*			
	−40 A to 20 A/	Solvent nature and concentration Cation nature (K^+^, Na^+^, Ca^2+^) Charge density of CP Swelling state of CP Temperature (−20 °C–115 °C)	“Beyond lithium-ion” batteries /Rechargeable; solid CP electrolyte, solid CP gel electrolyte; high energy density	Experimental: - Conductivity measurements (Turnkey Broadband Dielectric Spectrometer, Novocontrol Technologies, Montabaur, Germany); - SAXS spectra (Argonne APS Synchrotron beamline 12-ID-B); - Linear scan voltammetry
	−4 V to 3 V			
	for Na^+^ batteries (solvent DEG)			
	−5 A to 4 A/			
	−4 V to 6 V			
	for Ca^2+^ batteries (solvent DEG)			
	−160 A to 140 A/			
	−4 V to 3 V			
	for Na^+^ batteries (solvent EC:PC)	Polymer samples:1/4” (6.35 mm) diameter pieces		Ford &, 2020 [1]
	*Our method:*			
**PEGDMA-SS (1) + DCM or THF**	1.2 to 5.2 mA/cm^2^/0 V to 3.6 V	- System parameters: Conducting ions nature and radius, Solvent nature and concentration in SIPE, Energy density in the conversion electrode (correlated with the charge capacity and redox potential of the active species involved), Strain and stress in the polymeric electrolyte; - Parameters corresponding to the beads: the bead pair distance, bonds length, bond angles, and dihedral angles; choosing of the simulation mesh; - External parameters: electric field for polymeric gel testing, temperature.	similar	Structure description: (CGMD) model combined with the atomistic model; (DPD) model—for conductivity determination; Simulation (HFSS: Ansys HFSS 2022 R1, Mathcad: PTC Mathcad Prime 9)
	Correlated parameters: electric potential & ionic radius			
- for Na^+^ batteries: cathode: p-dopable polytriphenyl amine; anode: n-type redox-active poly(anthraquinonyl sulphide) or poly(anthraquinonyl imide)s (PAQIs); - for Mg^2+^ batteries: NTCDA-derived polyimide; - for Al^3+^ batteries: glassy carbon/Co_3_S_4_ cathode	0 to 4.6 mA/cm^2^/0 V to 3.9 V Correlated parameters: electric potential & solvent concentration			
		Electrode thickness = 400 μm, particles inside on the order of micrometers		
				
				
**Material: Electrolyte + Solvent**	**Current or Current Density [mA/cm^2^]/Obtained Electric Potential [V]**	**Control Parameters**	**Applications/Characteristics**	**Method of Determination/Source**
**/Conversion Electrodes**				
**PU-TFMSI (2) + (BzO)_2_ or THF**	*Reported in the literature:*			
		Swollen ratio	Flexible batteries and wearable devices; /Excellent mechanical performance; Electrochemical stability	Spectral analysis, H NMR spectra (Bruker AVANCE 400MHz III spectrometer, Brucker Optics, Leipzig, Germany); Infrared radiation spectra (Nicolet™ iS™ 10 FT-IR-Spectrometer—Thermo Fisher Scientific, Waltham, MA, USA); Mechanical tensile-stress (Instron 5944 Microtester, Instron, Norwood, MA, USA); Differential scanning calorimetry (Pyris 1 DSC—PerKin Elmer, Shelton, CT, USA); Thermogravimetric analysis (ASAP2020-Netzsch, Micromeritics, Norcross, GA, USA); X-ray Diffraction (X’TRA—Thermo Fisher Scientific, USA); SEM (Hitachi Model S-3400N Variable-Pressure SEM, Hitachi—Science & Technology, Berkshire, UK); X-ray photoelectron Spectrometry (PHI 5000 VersaProbe III, ULVAC-PHI, Inc., Hagisono, Chigasaki, Kanagawa, Japan)
		Temperature		
	0 to 0.7 mA/cm^2^/5 V to 6.5 V	PU-TFMSI membranes (2 mm × 35 mm) Cathode (LFP)/Li anode		
				Cai &, 2022 [11,15]
	*Our method:*			
**PU-TFMSI (2) + (BzO)_2_ or THF**	0.1 to 5.1 mA/cm^2^/0 V to 4.3 V	- System parameters: Conducting ions nature and radius, Solvent nature and concentration in SIPE, Energy density in the conversion electrode (correlated with the charge capacity and redox potential of the active species involved), Strain and stress in the polymeric electrolyte; - Parameters corresponding to the beads: the bead pair distance, bonds length, bond angles, and dihedral angles; choosing of the simulation mesh; - External parameters: electric field for polymeric gel testing, temperature.	similar	Structure description: (CGMD) model combined with the atomistic model; (DPD) model—for conductivity determination; Simulation (HFSS: Ansys HFSS 2022 R1, Mathcad: PTC Mathcad Prime 9)
	Correlated parameters: electric potential & ionic radius			
- for Na^+^ batteries: cathode: p-dopablepolytriphenylamine; anode: n-type redox-active poly(anthraquinonylsulphide) or poly(anthraquinonylimide)s (PAQIs); - for Mg^2+^batteries: NTCDA-derived polyimide; - for Al^3+^ batteries: glassy carbon/Co_3_S_4_ cathode	0 to 4.95 mA/cm^2^/0 V to 4.25 V Correlated parameters: electric potential & solvent concentration			
		Electrode thickness = 400 μm, particles inside on the order of micrometers		
				
				
**Material: Electrolyte + Solvent**	**Current or Current Density [mA/cm^2^]/Obtained Electric Potential [V]**	**Control Parameters**	**Applications/Characteristics**	**Method of Determination/Source**
**/Conversion Electrodes**				
**PDMS-poly(MPA-Na^+^-*r*-PEGMA)_20_-PDMS_10_ (3) + DMF or THF**	*Reported in the literature:*			
	1.6 to 2.3 mA/cm^2^/3.8 V to 2.5 V	Monomer nature	Stretchable batteries/electronics Stretchable functional polymeric materials /Elastic SIPEs	Spectral analysis, H NMR spectra (Bruker AVANCE 400MHz III spectrometer, Brucker Optics, Leipzig, Germany); SEM (Hitachi Model S-3400N Variable-Pressure SEM, Hitachi—Science & Technology, Berkshire, UK); Energy Dispersive X-ray Spectroscopy (Spectra 200 TEM, Thermo Fisher Scientific Inc., Waltham, MA USA); Differential scanning calorimetry (Pyris 1 DSC—PerKin Elmer, Shelton, CT, USA); Broadband dielectric spectroscopy (BDS) (Novocontrol BDS Concept 80, Novocontrol Technologies, Montabaur, Germany) Galvanostatic test Gel Permeation Chromatography (GPC) (1260 Infinity II LC System, Agilent, Santa Clara, CA, USA) Atomic adsorption spectroscopy (AAS) (Routine Analyzer novAA 800, Analytik Jena, Jena, Germany)
		Molar ratio of PDMS and grafted block copolymers		
		Temperature		
		Frequency		
		Polymer membranes (5.0 mm × 4.0 cm)		
				Cao &, 2020 [18]
	*Our method:*			
**PDMS-poly(MPA-Na^+^-*r*-PEGMA)_20_-PDMS_10_ (3) + DMF or THF**	1.9 to 5.8 mA/cm^2^/0 V to 2.8 V Correlated parameters: electric potential & ionic radius	- System parameters: Conducting ions nature and radius, Solvent nature and concentration in SIPE, Energy density in the conversion electrode (correlated with the charge capacity and redox potential of the active species involved), Strain and stress in the polymeric electrolyte; - Parameters corresponding to the beads: the bead pair distance, bonds length, bond angles, and dihedral angles; choosing of the simulation mesh; - External parameters: electric field for polymeric gel testing, temperature.	similar	Structure description: (CGMD) model combined with the atomistic model; (DPD) model—for conductivity determination; Simulation (HFSS: Ansys HFSS 2022 R1, Mathcad: PTC Mathcad Prime 9)
	Correlated parameters: electric potential & ionic radius			
- for Na+ batteries: cathode: p-dopablepolytriphenylamine; anode: n-type redox-active poly(anthraquinonylsulphide) or poly(anthraquinonyl imide)s (PAQIs); - for Mg^2+^ batteries: NTCDA-derived polyimide; - for Al^3+^ batteries: glassy carbon/Co_3_S_4_ cathode	0 to 3.5 mA/cm^2^/0 V to 3 V Correlated parameters: electric potential & solvent concentration			
		Electrode thickness = 400 μm, particles inside on the order of micrometers		

## Data Availability

Data are contained within the article.
